# Migration and aggregation of Pt atoms on metal oxide-supported ceria nanodomes control reverse water gas shift reaction activity

**DOI:** 10.1038/s42004-023-01064-4

**Published:** 2023-12-05

**Authors:** Haodong Wang, Ryuichi Shimogawa, Lihua Zhang, Lu Ma, Steven N. Ehrlich, Nebojsa Marinkovic, Yuanyuan Li, Anatoly I. Frenkel

**Affiliations:** 1 Department of Materials Science and Chemical Engineering, Stony Brook University, Stony Brook, NY 11794 USA; 2 Mitsubishi Chemical Corporation, Science & Innovation Center, 1000, Kamoshida-cho, Aoba-ku, Yokohama, 227-8502 Japan; 3 Center for Functional Nanomaterials, Brookhaven National Laboratory, Upton, NY 11973 USA; 4 National Synchrotron Light Source II, Brookhaven National Laboratory, Upton, NY 11973 USA; 5 Department of Chemical Engineering, Columbia University, New York, NY 10027 USA; 6 Chemical Sciences Division, Oak Ridge National Laboratory, Oak Ridge, TN 37831 USA; 7 Chemistry Division, Brookhaven National Laboratory, Upton, NY 11973 USA

**Keywords:** Heterogeneous catalysis, Nanoparticles, Nanoparticles, Surface spectroscopy, Catalyst synthesis

## Abstract

Single-atom catalysts (SACs) are particularly sensitive to external conditions, complicating the identification of catalytically active species and active sites under in situ or operando conditions. We developed a methodology for tracing the structural evolution of SACs to nanoparticles, identifying the active species and their link to the catalytic activity for the reverse water gas shift (RWGS) reaction. The new method is illustrated by studying structure-activity relationships in two materials containing Pt SACs on ceria nanodomes, supported on either ceria or titania. These materials exhibited distinctly different activities for CO production. Multimodal operando characterization attributed the enhanced activity of the titania-supported catalysts at temperatures below 320 ˚C to the formation of unique Pt sites at the ceria-titania interface capable of forming Pt nanoparticles, the active species for the RWGS reaction. Migration of Pt nanoparticles to titania support was found to be responsible for the deactivation of titania-supported catalysts at elevated temperatures. Tracking the migration of Pt atoms provides a new opportunity to investigate the activation and deactivation of Pt SACs for the RWGS reaction.

## Abstract (web summary)

Platinum dispersed on metal oxide supports is widely used for industrially important catalysis such as the reverse water gas shift reaction, but active site migration and subsequent alterations in catalytic performance are still not fully understood. Here, the use of platinum on ceria nanodomes shows the detrimental effect of migration of platinum nanoparticles to titania supports at elevated temperatures.

## Introduction

Single-atom catalysts (SACs) have been extensively investigated due to their efficient use of precious metals as well as distinct activity and selectivity^1–3^. Their tendency towards aggregation in reaction conditions often leads to the coexistence of atomically dispersed species and nanoparticles, which can result in migration of active species and severely limits identification of catalytically active species and active sites^4–8^. In this work, we take on the task of tracking the structural evolution of SACs to nanoparticles for the reverse water gas shift (RWGS) reaction that has been widely investigated as a means of converting greenhouse gas CO_2_ to CO, which can be used as building blocks to generate valuable chemicals and fuels for industrial use^9–12^. Pt catalysts are widely utilized for the RWGS reaction, especially for those dispersed on reducible metal oxide support, which can provide abundant oxygen vacancies for CO_2_ absorption and activation^13–17^. However, atomically dispersed Pt species can undergo aggregation under reaction conditions, leading to the migration of active sites and subsequent alterations in catalytic performance^8,18,19^. The catalytic activity of supported Pt catalysts can be influenced by the coordination environment of Pt species and the CO adsorbates attached to Pt species (CO poisoning) under the RWGS reaction^8,20,21^. Recent studies have demonstrated that atomically dispersed Pt is capable of high CO selectivity while large Pt nanoparticles tend to produce CH_4_ production^22,23^. However, it is still under debate whether Pt atoms can remain atomically dispersed at elevated temperatures under reduction conditions^4,7,24^. Previously, we employed ceria nanodomes supported on ceria or titania support to achieve high Pt weight loadings and found that Pt locations can be tuned on ceria support, ceria-titania interface or titania support^25,26^. The modified metal-support interaction and electronic properties of Pt single atoms ultimately guide their catalytic activity in reaction conditions.

In this study, we introduce a well-defined system consisting of Pt single atoms supported on ceria nanodomes. The nanodomes are supported, in one system, on ceria, and, in the second one, on titania, which enables a direct comparison between the catalytic activities of Pt species with distinct coordination environments, as well as an exploration of the impact of CO poisoning on these locations. We hypothesize that the Pt catalysts will exhibit different activities for the RWGS reaction due to the migration and aggregation of the Pt species on the two distinct supports. Recent studies show that incorporating TiO_2_ in addition to CeO_2_ support could enhance the reducibility and oxygen mobility on CeO_2_ support, and unique sites could be formed at the interface of CeO_2_-TiO_2_^27–30^. In order to better understand the speciation and structure of different types of supported Pt catalysts, we employed several structural characterization techniques, including scanning transmission electron microscopy (STEM), diffuse reflectance infrared Fourier transform spectroscopy (DRIFTS), and X-ray absorption spectroscopy (XAS). Our focus was on examining the evolution of the coordination environment and electronic states of Pt species throughout the RWGS reaction. The results demonstrated the utility of a combined study for tracking different species coexisting in catalytic ensembles in reaction conditions and complementary investigation of both metal-support and metal-adsorbate interaction as well as their effects on catalytic performance in presence of heterogeneously distributed metal species.

## Results and discussion

### Catalytic performance and ex situ characterization

**Fig. 1:**
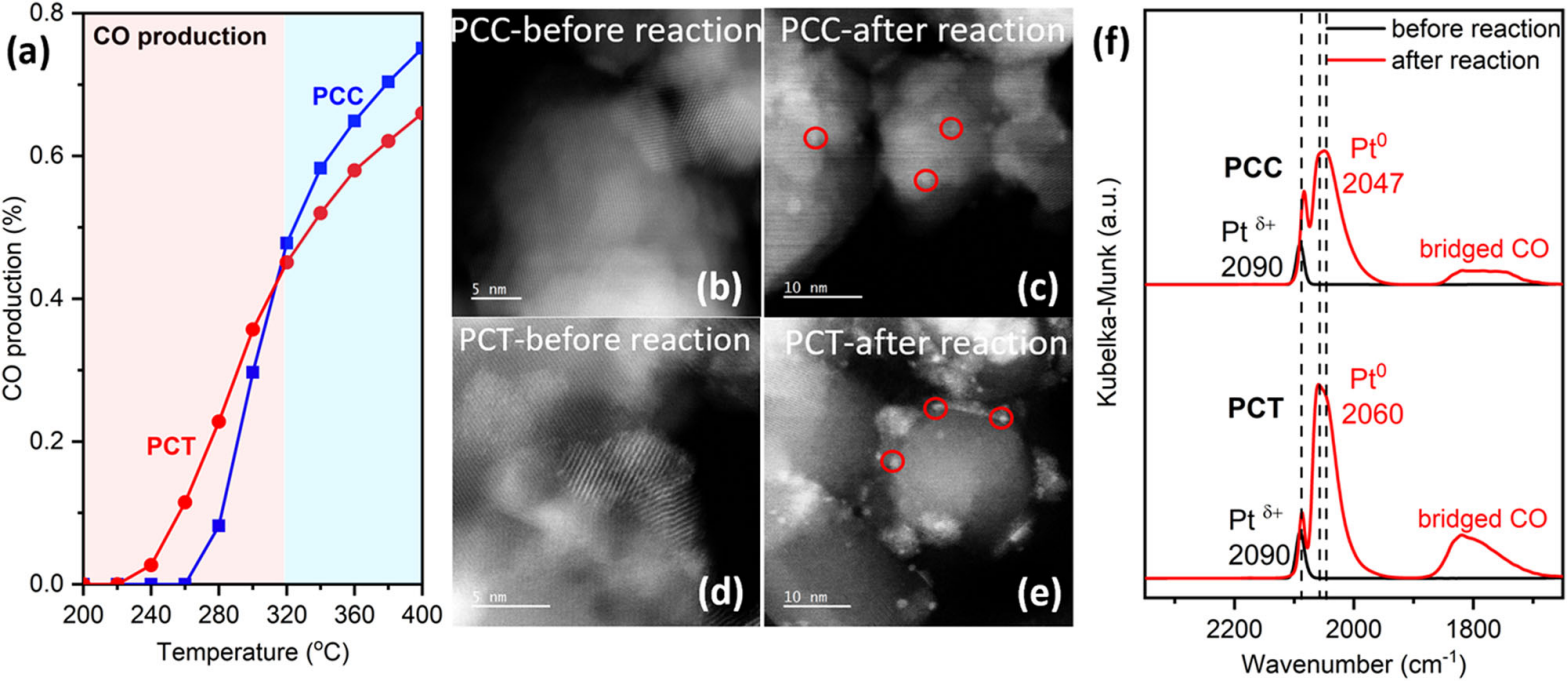
Pt-nanoceria-CeO_2_ (PCC) vs. Pt-nanoceria-TiO_2_ (PCT), CO production during the RWGS reaction and ex situ characterization before and after reaction. **a** CO production of PCC vs. PCT during the RWGS reaction; **b**–**e** STEM images show aggregation of Pt atoms after reaction in both samples but not in the as-prepared samples. (Figure S2 (a) and (b) indicate both samples have similar particle sizes after the reaction); **f** CO-probed DRIFTS after CO desorption of PCC vs. PCT before reaction vs. after reaction.

The two Pt catalysts were synthesized by a wet impregnation method, resulting in Pt weight loadings of 0.94 wt.% for Pt-nanoceria-CeO_2_ (labeled as PCC) sample and 0.95 wt.% for Pt-nanoceria-TiO_2_ (PCT) sample, determined by ICP-OES analysis. The RWGS reaction was carried out from room temperature to 400 °C under a gas mixture of 5% CO_2_ and 5% H_2_ balanced in He with a total flow rate of 31.5 ml/min. Both samples exhibit remarkable CO selectivity (>96%) throughout the entire reaction as shown in Fig. S1. The temperature dependent CO productions of two samples are illustrated in Fig. 1a. Two catalysts show distinct initiation temperatures for the reaction: 280 °C for the PCC sample and 240 °C for the PCT sample. Up to 300 °C, the PCT sample exhibits superior catalytic activity compared to the PCC sample, while as the temperature reaches 320 °C and above, the PCC sample emerges as the frontrunner. The contrasting catalytic behavior of the two samples implies that Pt species in these two catalysts experience different structural evolutions during the reaction.

To reveal the size change of Pt species in these two catalysts, STEM images were collected. Figure 1b, d indicate no evidence of Pt nanoparticles in the as-prepared samples. However, upon examining the spent catalytic samples, small nanoparticles were observed in both the PCC and PCT samples, as indicated by the red circles in Fig. 1c, e. Based on the statistical analysis of distributed particles (Fig. S2), the average particle size for PCC samples is 1.4 nm and for PCT sample 1.3 nm. For the PCT sample, Pt nanoparticles were formed on CeO_2_, interface of CeO_2_-TiO_2_ and TiO_2_. To complement the STEM technique, which is limited by resolution and Z-contrast, CO-probed DRIFTS was performed on the as-prepared and spent catalysts. For both as-prepared samples, DRIFTS spectra feature a single, narrow and symmetric peak at 2090 cm^−1^ (Fig. 1f), which corresponds to CO linearly absorbed on top of a single Pt site with positive charge (Pt ^δ+^)^1,31,32^. According to our previous findings, the observed frequency of 2090 cm^−1^ is consistent with Pt located on the surface of CeO_2_ support^25,26^. After the RWGS reaction, three distinct peaks were observed in both spent samples. In Fig. 1f, the leftmost red sharp peaks which are similar to the ones shown in fresh samples correspond to CO binding to Pt single sites. The relatively broad peaks in the middle, measured at 2047 cm^−1^ for PCC and 2060 cm^−1^ for PCT, indicate higher coordinated sites of metallic Pt nanoparticles^20,31,33^. Based on these two observations, the ratio of Pt nanoparticles to Pt single atoms in the spent PCC sample was determined to be 88% and in PCT was 97% through quantitative DRIFTS calculations shown in Fig. S2^8,20,34^. Furthermore, it was observed that the broad peaks near 1800 cm^-1^ correspond to bridged CO sites^31^. It is interesting to observe two samples exhibiting similar trends of aggregation of Pt nanoparticles after the RWGS reaction, transforming from single atoms to small nanoparticles of similar size, yet displaying varying catalytic activity during the reaction. This discrepancy raises the question: *why the activity of two catalysts varies below and above 320 ˚C?* To gain insight into the structural evolution underlying the observed differences in activity, we conducted in situ DRIFTS and XAS experiments under reaction conditions.

### Structure evolution of Pt atoms by in situ characterization

**Fig. 2:**
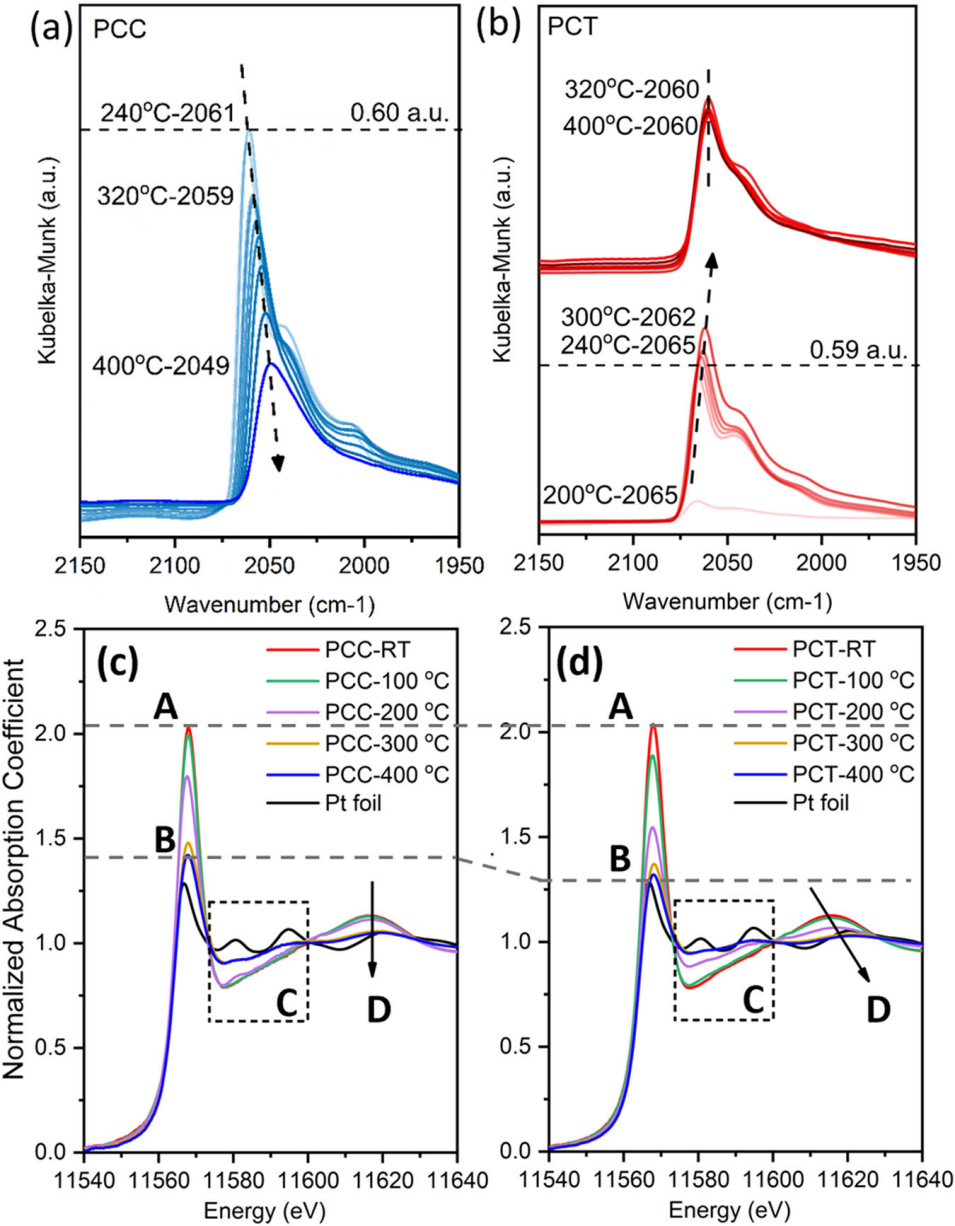
In situ DRIFTS and XAS spectra of PCC vs. PCT. DRIFTS spectra of PCC (**a**) and PCT (**b**) samples under the RWGS condition from RT to 400 °C; Pt L_3_ edge XAS spectra during the RWGS reaction (**c**) PCC; (**d**) PCT. Pt foil spectrum is also included as reference.

In situ DRIFTS results of two samples are presented in Fig. 2a, b. Since no CO was fed in the experiment, the observed CO-Pt contribution arose from the product of the RWGS reaction. In the PCC sample, the CO-Pt peak emerges at 2061 cm^−1^ at 240 °C, while in contrast, it appears at 200 °C in the PCT sample at 2065 cm^−1^. At 240 °C, both samples exhibit comparable intensity and position, indicating that Pt remains in similar locations at the beginning of the reaction. Furthermore, no contribution of the Pt single atom features was observed in either sample, suggesting that Pt single atoms disappear under reaction conditions or the CO-Pt single atom feature is too weak to detect compared to the CO-Pt nanoparticle. As the reaction progresses, for the PCC sample, the CO-Pt contribution continuously shifts towards the lower wavenumbers and decreases in intensity, suggesting a continuous change in the coordination environment of Pt in the PCC sample. In contrast, the intensity of the CO-Pt peak in the PCT sample continues to increase until 320 °C, after which it remains constant in both intensity and wavenumber. The different trends shown before and after 320 °C indicate that the PCT sample experienced a drastic structural change.

To further understand why the Pt-CO contribution in the PCT sample stabilizes above 320 °C and to explore the relationship between structural changes and activity, in situ XAS experiments were conducted under reaction conditions. The Pt L_3_ XANES spectra are shown in Fig. 2c, d. At room temperature, both samples exhibit comparable white line intensity (region A). During the RWGS reaction, the reduction of Pt species occurs faster in the PCT sample, as evidenced by the gradual decrease in white line intensity (region B) compared to the PCC sample. In the post-edge (region C), the PCC sample shows minimal changes until 300 °C, whereas the PCT sample indicates an intermediate status at 200 °C. This phenomenon is also evident in region D. For the PCT sample, the feature in region D shifts to a higher energy range when the temperature goes above 300 °C, while it’s not observed in the PCC sample. The differences observed between the two samples align with the findings from DRIFTS results, indicating a significant structural evolution in the PCT sample after reaching 300 °C. These intriguing results from the examination of XAS data have motivated us to perform further XANES analysis and EXAFS fitting, which will allow us to gain a deeper understanding of the electronic states and coordination environment changes of the two samples during the reaction.

**Fig. 3:**
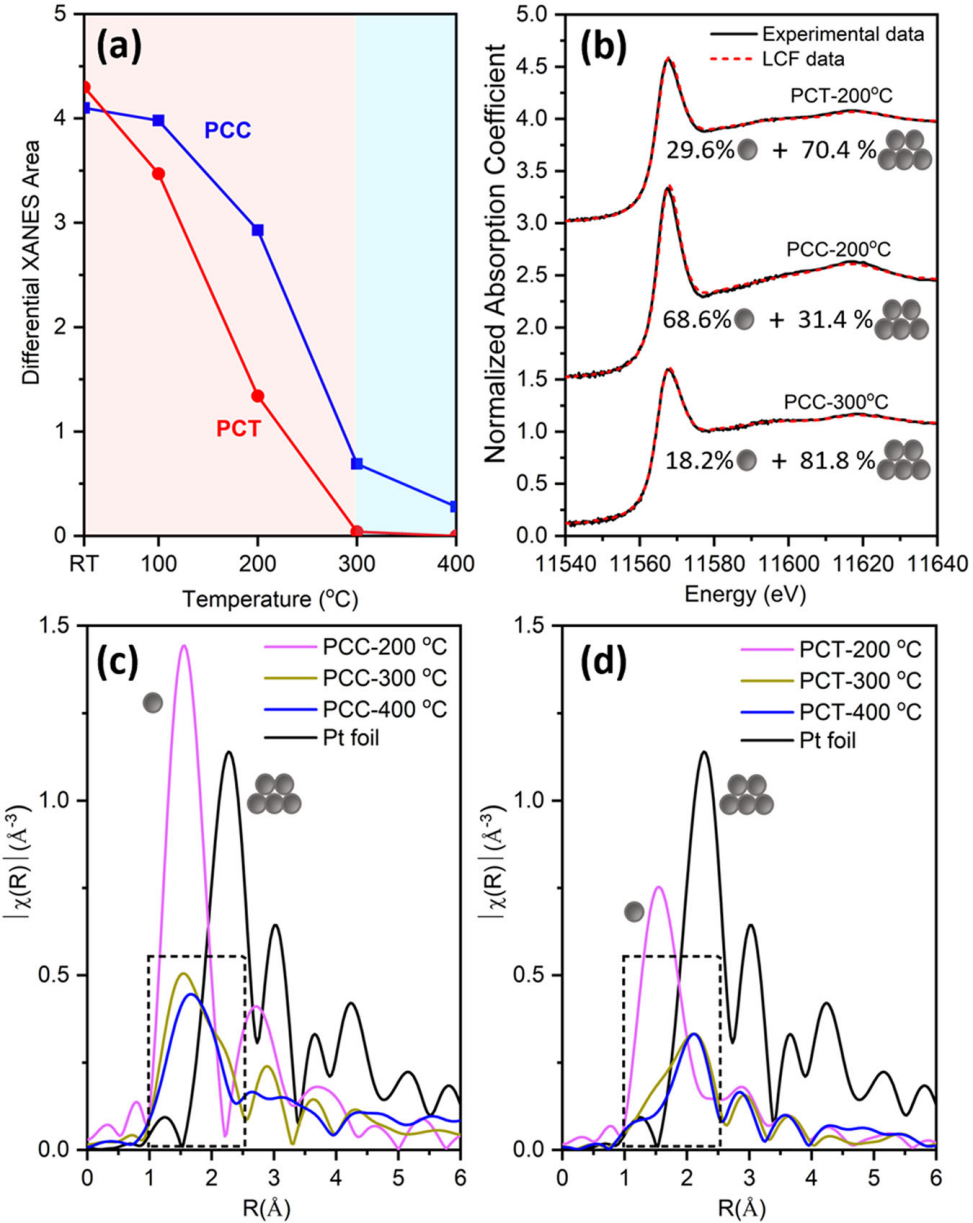
XANES and EXAFS data analysis. **a** Differential XANES area of PCC vs. PCT using PCT-400 °C data as reference; **b** Comparison of experimental data vs. linear combination fitting (LCF) results using PCT-RT and PCT-300 °C data as reference; Fourier transform magnitudes of k^2^-weighted Pt L_3_ EXAFS spectra (**c**) PCC samples; (**d**) PCT samples.

Based on the XAS data examination above, XANES differential area analysis and linear combination fitting were performed, and the results are shown in Fig. 3a, b, respectively. From room temperature to 300 °C, the greater change in the XANES area of the PCT sample indicates that the average electronic density of the Pt species changes more rapidly than that of the PCC sample. Above 300 °C, minimal changes were observed in the PCT sample, which is consistent with DRIFTS results. For the linear combination fitting, the PCT room temperature XAS data, which indicates a Pt single atom structure, and the PCT-300 °C XAS data, indicating a Pt nanoparticle structure as determined by EXAFS fitting (Fig. S4 and Table S1), were used as references. The results presented in Fig. 3b reveal that approximately 70% of the Pt species in the PCT sample formed nanoparticles at 200 °C, whereas only 31% did so in the PCC sample. At 300 °C, nearly 20% of the Pt species in the PCC sample remains as single atoms. The Fourier transform magnitudes of k^2^-weighted EXAFS spectra are shown in Fig. 3c, d. The region outlined by the dashed rectangle indicates that reduction of Pt-O-Ce was still occurring in the PCC sample above 300 °C, while the Pt species were already fully reduced to Pt-Pt coordinated nanoparticles in PCT sample at 300 °C, as evidenced by the first shell fitting results shown in Figs. S5 and S6. These findings, correlated with DRIFTS results, demonstrate ongoing changes in the PCC sample above 300 °C, and steady state conditions for the PCT sample.

### Activation and deactivation of Pt species

**Fig. 4:**
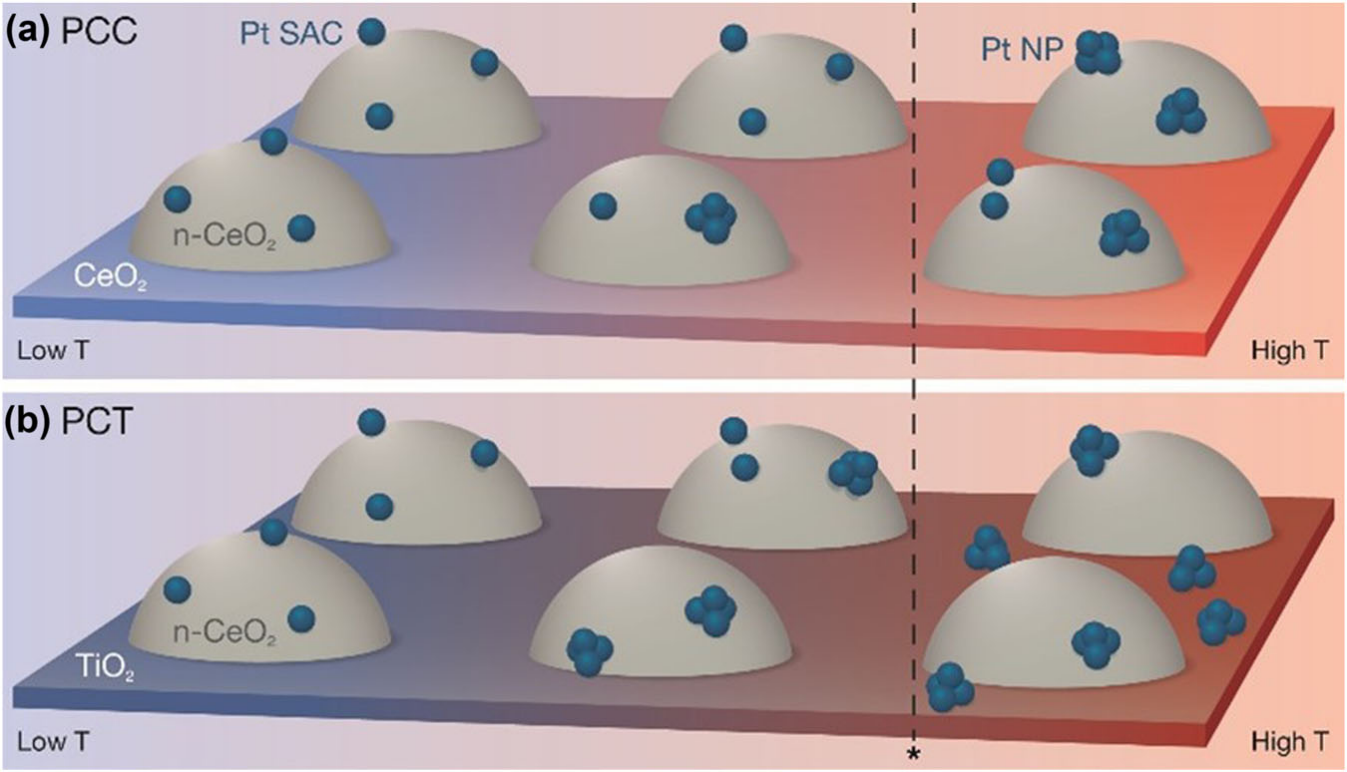
Scheme of proposed structural changes during the RWGS reaction. The three temperatures (from left to right) are room temperature, 300 °C and 400 °C. **a**: Pt-ceria-CeO_2_ (PCC); **b**: Pt-ceria-TiO_2_ (PCT).

According to the catalytic activity test and in situ structure characterizations using DRIFTS and XAS, we proposed the structural changes of two samples during the RWGS reaction, as illustrated in Fig. 4. From room temperature to 300 °C, the reaction rate was mainly dominated by the reduction of the Pt-O-Ce bond. The PCT sample exhibited higher activity due to its more intense reduction, leading to undercoordinated Pt atoms and formation of Pt nanoparticles at the ceria-titania interface. At elevated temperatures, Pt species in the PCT sample migrated to TiO_2_ support, as indicated by DRIFTS and XAS results. In the EXAFS fitting of the PCT 300 °C data, Pt-CO multiple-scattering paths were included in the fit model to accurately capture the feature observed in the range of 1–1.5 Å (marked within the dashed line), which was not observed in the PCC sample. In addition, by comparing the DRIFTS spectra under reaction conditions, we observed a significant difference in the CO-Pt bond strength, which decreased drastically with increasing temperatures for the PCC sample. By contrast, for the PCT sample at elevated temperatures, the CO-Pt bond intensity indicated minimum changes. The strong bonding between CO and Pt atoms results in CO poisoning, wherein the surface becomes saturated with CO and inhibits further CO production in the PCT sample, as evidenced by both EXAFS and DRIFTS results. Previous studies have shown that CO_2_ dissociation which can heal oxygen vacancies in the support was determined by the oxygen mobility of the support^21,23,35^. Compared to CeO_2_ support, TiO_2_ support is less effective for the binding and dissociation of reactant^36^. In summary, our in situ DRIFTS and XAS spectra exhibit that the dispersion of Pt species to TiO_2_ and the strong Pt-CO bond strength lead to the deactivation of the PCT sample, while Pt-O-Ce bond reduction remains active in the PCC sample, as shown in DRIFTS and XAS, leading to enhanced activity at 400 °C.

The proposed evolution of the supported Pt catalyst structure offers promising prospects for the development of efficient catalysts. The findings highlight that Pt catalysts can achieve high activity when they are undercoordinated, but their performance may decline once the location of Pt species changes. Additionally, the Pt-CO adsorption effect also plays a significant role in the catalytic activity of the RWGS. Therefore, it is essential to focus on both metal-support interaction and metal-adsorbate interaction to optimize catalyst performance.

## Methods

### Sample preparation

All samples were prepared by dispersing 0.5 g cerium (IV) oxide nanopowder (<25 nm) or titanium (IV) oxide nanopowder (<25 nm) in a solution of 0.42 g of cerium (III) nitrate hexahydrate, 2.0 g urea and 8 mL of water. The weight loading of Pt was controlled by adding the desired amount of Pt precursor solution (chloroplatinic acid hydrate in water, approximately 1 wt.% Pt) in each system. All chemicals were purchased from Sigma-Aldrich. Ultrapure water was provided by a Millipore purification system. The mixture was sealed in a glass vial and stirred for 24 hours in an oil bath at 90 °C. Afterward, the samples were washed with DI water and centrifuged three times. After drying overnight in an oven at 80 °C, the samples were crushed into powders and calcined at 500 °C (5 °C/min heating ramp) for 5 h. The resulting platinum loadings of all samples were measured by inductively coupled plasma optical emission spectrometry (ICP-OES) by Galbraith Laboratories. The instrument measures characteristic emission spectra by optical spectrometry and describes multi-elemental determinations.

### Scanning transmission electron microscope (STEM)

Ex situ electron microscopy was performed at the Center for Functional Nanomaterials at Brookhaven National Laboratory on a Hitachi 2700D scanning transmission electron microscope equipped with a probe aberration corrector operated at 200 kV.

### Diffuse reflectance infrared Fourier transform spectroscopy (DRIFTS)

The spectra were collected using a Thermo-Nicolet iS50 FTIR spectrometer equipped with a rapid-scanning option, liquid-nitrogen-cooled mercury cadmium telluride (MCT) detector, and a Praying Mantis High Temperature Reaction Chamber (Harrick Scientific Products). Prior to measurement, each sample was heated at 150 °C for 30 min under He with a flow rate of 20 ml/min to remove surface adsorbed species. Afterward, the background spectrum was collected under He with a flow rate of 20 ml/min at room temperature. For the ex situ measurement, a mixture of 10% CO (balanced with He) with a flow rate of 20 ml/min flowed through the reaction chamber for 30 min to acquire CO adsorption spectra, followed by flowing He with a flow rate of 20 ml/min for 30 min to acquire CO desorption spectra. For the in situ measurement under reaction conditions, 5% CO_2_ and 5% H_2_ (balanced with He; total flow rate of 31.5 ml/min) traveled through the sample.

### X-ray absorption spectroscopy (XAS)

Pt L_3_-edge XAS measurements were performed at the QAS beamline (7-BM) of the National Synchrotron Light Source II (NSLS II), Brookhaven National Laboratory. For ex situ measurements, samples were prepared as pellets using a hydraulic press. For each sample, 30 scans, each of 30 sec durations, were collected in fluorescence mode to increase the signal-to-noise ratio. For the in situ reactions, the gas mixture of 5% CO_2_, 5% H_2_ and 90% He with the total flow rate of 31.5 ml/min was purging the sample.

### Catalytic activity test

The as-prepared catalyst was loaded in a quartz tube (2 mm I.D., 2.4 mm O.D.) and mounted in a Clausen cell. For the reaction, 5% CO_2_ and 5% H_2_ (balanced with He; total flow rate of 31.5 ml/min) traveled through the sample. The activity test was executed by a stepwise increase of the reaction temperature from room temperature (RT) to 400 °C. The reactants and products were analyzed by a mass spectrometer (Hiden QGA).

### Supplementary information

Supplementary Information

## Data Availability

The data supporting the findings of this study are available within the article and its Supplementary Information file. Additional information and figures are provided in the Supplementary Information file. Other data are available from the authors upon request.
