# Supplementary material for: Migration and aggregation of Pt atoms on metal oxide-supported ceria nanodomes control reverse water gas shift reaction activity
Source: Commun Chem. 2023 Dec 5;6:264. doi: 10.1038/s42004-023-01064-4 (PMC10697936; doi:10.1038/s42004-023-01064-4)
Supplement: Supplementary file 1 — Supplementary Information [file 42004_2023_1064_MOESM1_ESM.pdf]

Supplementary Information

Migration and aggregation of Pt atoms on metal oxide-supported ceria nanodomes control reverse water gas shift reaction activity

Haodong Wang,[1] Ryuichi Shimogawa,[1,2] Lihua Zhang,[3] Lu Ma,[4] Steven N. Ehrlich,[4] Nebojsa Marinkovic,[5] Yuanyuan Li,[6], and Anatoly I. Frenkel[1,7*]*

1 Department of Materials Science and Chemical Engineering, Stony Brook University, Stony Brook, NY 11794, United States

2 Mitsubishi Chemical Corporation, Science & Innovation Center, 1000, Kamoshida-cho, Aoba-ku, Yokohama 227-8502, Japan

3 Center for Functional Nanomaterials, Brookhaven National Laboratory, Upton, NY 11973, United States

4 National Synchrotron Light Source II, Brookhaven National Laboratory, Upton, New York 11973, United States

5 Department of Chemical Engineering, Columbia University, New York NY 10027, United States

6 Chemical Sciences Division, Oak Ridge National Laboratory, Oak Ridge, TN 37831, United States

7 Chemistry Division, Brookhaven National Laboratory, Upton, NY 11973, United States

Table of Contents

1) Figure S1. CO selectivity during the RGWS reaction over PCC and PCT samples.
2) Figure S2. Particle size distributions of used PCC and PCT samples.
3) Figure S3. Quantification of DRIFTS spectra by peak deconvolution and integration.
4) Figure S4. Comparison between experimental spectra and EXAFS fit spectra of the PCT sample at room temperature vs. at 300 ˚C under reaction condition.
5) Figure S5. Comparison of experimental spectra and EXAFS fit spectra for the first shell Pt-O bonds in the two samples.

6) Figure S6. Summary of Pt-O coordination number changes and bond length variations.
7) Table S1. EXAFS fit results of the PCT sample at room temperature and at 300 ˚C under reaction condition.

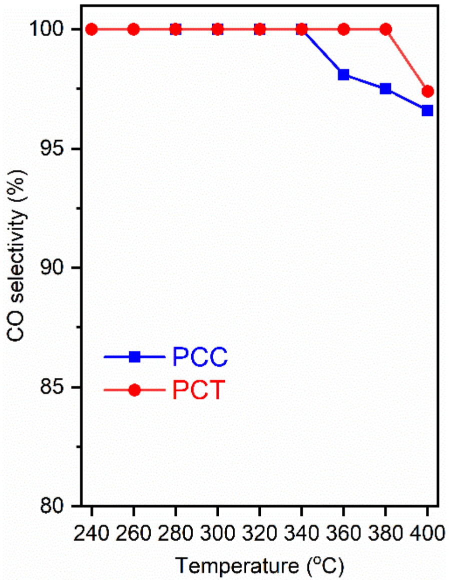

Figure S1. CO selectivity during the RGWS reaction over PCC and PCT samples.

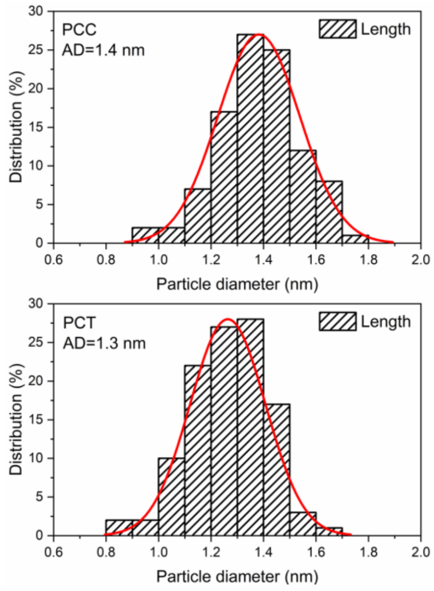

Figure S2. Particle size distributions of used PCC and PCT samples.

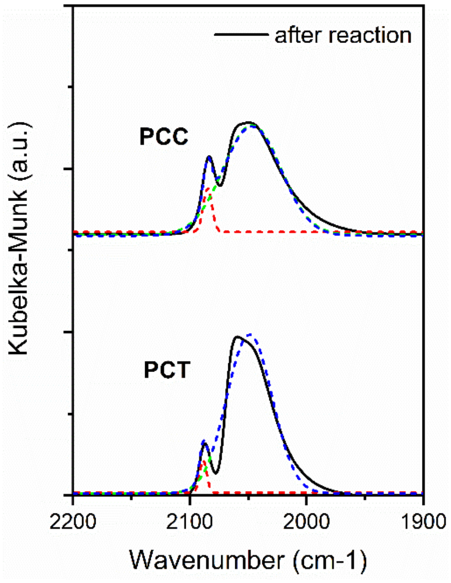

Figure S3. Quantification of DRIFTS spectra by peak deconvolution and integration.

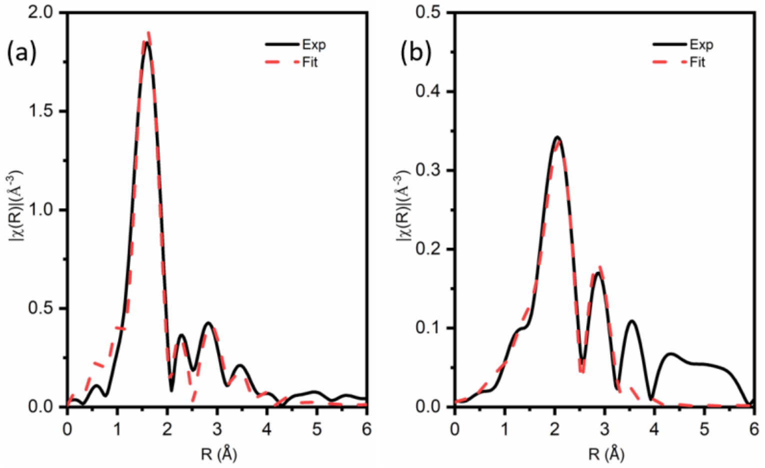

Figure S4. Comparison between experimental spectra and EXAFS fit spectra of the PCT sample (a) at room temperature vs. (b) at 300 ˚C under reaction condition. k range is 2.5-9.0 Å-1 and R range is 1.3-3.3 Å.

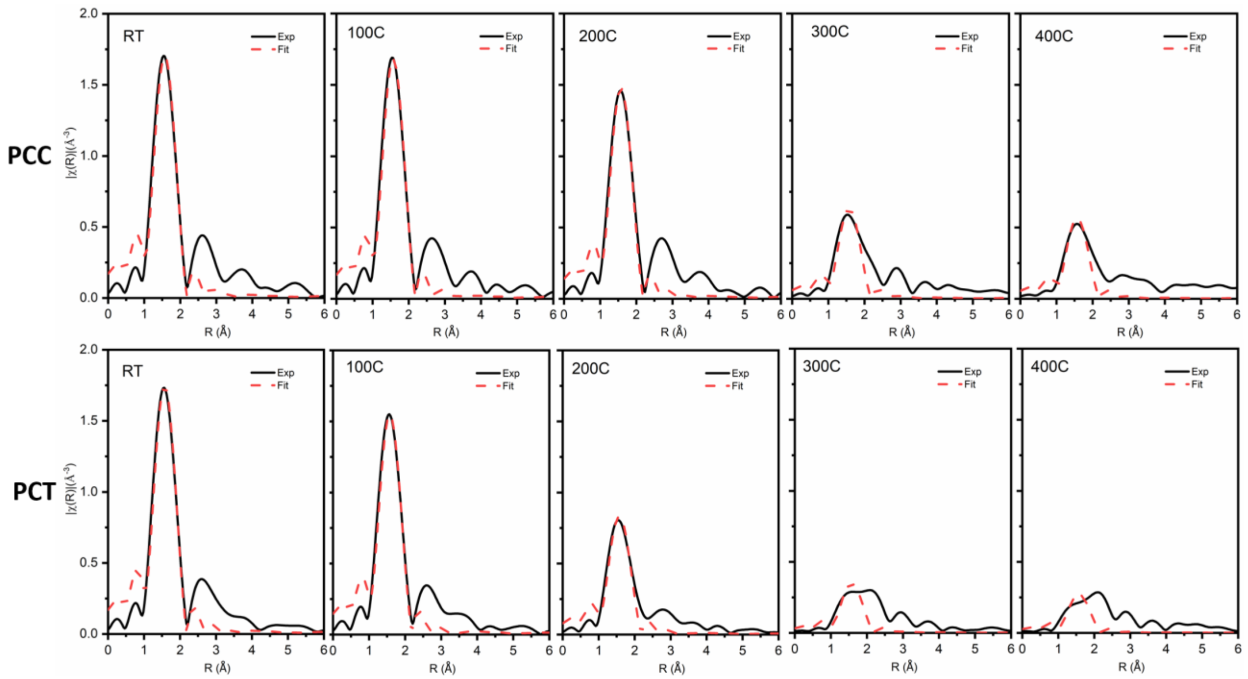

Figure S5. Comparison of experimental EXAFS data and first shell (Pt-O) fits in the two samples. k range is 2.5-8.0 $Å^{-1}$ and R range is 1.3-2.3 Å.

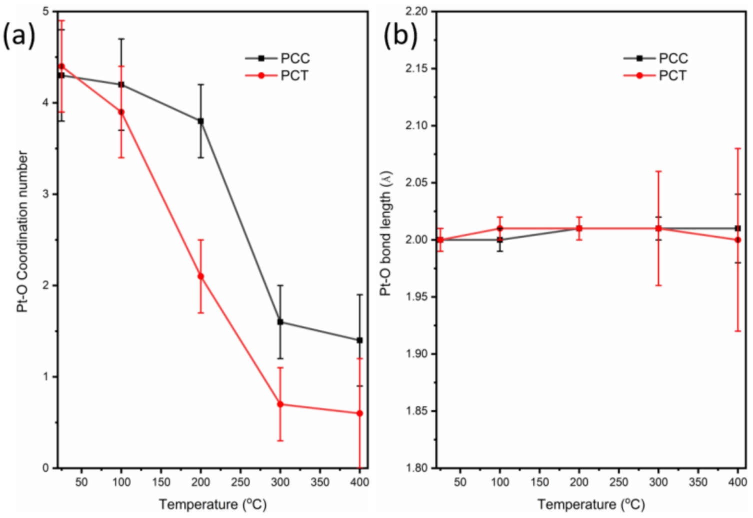

Figure S6. Pt-O coordination number changes and bond length variations.

Table S1. EXAFS fit results of the PCT sample at room temperature and at 300 ˚C under reaction condition. k range is 2.5-9.0 Å^{-1} and R range is 1.3-3.3 Å. Uncertainties in the last significant digits are given in parentheses.

Temperature	Path	Coordination number	Radial distance (Å)
**RT**	Pt-O	4.5(6)	2.00(1)
	Pt-Ce	1.4(7)	3.45(3)
	Pt-O	5.5(1.6)	3.50(3)
**300 ˚C**	Pt-C	0.58(2)	1.94(4)
	Pt-Pt	7.1(1.8)	2.68(1)
	Pt-O	0.58	3.04(4)
	Pt-C-O	0.58	3.04(4)
	Pt-C-O-C	0.58	3.04(4)
